# Molecular mechanism of Hedyotis Diffusae Herba in the treatment of lupus nephritis based on network pharmacology

**DOI:** 10.3389/fphar.2023.1118804

**Published:** 2023-06-08

**Authors:** Jinfei Yang, Siying Li

**Affiliations:** ^1^ Department of Dermatology, Hunan Key Laboratory of Medical Epigenomics, The Second Xiangya Hospital of Central South University, Changsha, Hunan, China; ^2^ Department of Nephrology, Hunan Key Laboratory of Kidney Disease and Blood Purification, The Second Xiangya Hospital of Central South University, Changsha, Hunan, China

**Keywords:** Hedyotis Diffusae Herba, network pharmacology, lupus nephritis, TNF, VEGFA, bioactive components

## Abstract

**Aims:** To determine the bioactive components of Hedyotis Diffusae Herba (HDH) and the targets in treating lupus nephritis (LN), and so as to elucidate the protective mechanism of HDH against LN.

**Methods and results:** An aggregate of 147 drug targets and 162 LN targets were obtained from online databases, with 23 overlapped targets being determined as potential therapeutic targets of HDH against LN. Through centrality analysis, TNF, VEGFA and JUN were screened as core targets. And the bindings of TNF with stigmasterol, TNF with quercetin, and VEGFA with quercetin were further validated by molecular docking. By conducting Kyoto Encyclopedia of Genes and Genomes (KEGG) and Gene Ontology (GO) enrichment analyses for drug targets, disease targets and the shared targets, TNF signaling pathway, Toll-like receptor signaling pathway, NF-kappa B signaling pathway and HIF-1 signaling pathway, etc., were found in all these three lists, indicating the potential mechanism of HDH in the treatment of LN.

**Conclusion:** HDH may ameliorate the renal injury in LN by targeting multi-targets and multi-pathways, including TNF signaling pathway, NF-kappa B signaling pathway, HIF-1 signaling pathway and so on, which provided novel insights into further researches of the drug discovery in LN.

## Introduction

Systemic lupus erythematosus (SLE) is a multi-system autoimmune disease along with abnormally activated immune system, attacking practically any organ system in the human body (Calle-Botero and Abril, 2020; Kiriakidou and Ching, 2020). In SLE, lupus nephritis (LN) is considered as the major pathogenic and fatal risk contributors (Maria and Davidson, 2020), affecting almost 40% of adults with SLE, with 10% of LN patients having to face the torment of end-stage renal disease ultimately (Bastian et al., 2002; Jakes et al., 2012; Almaani et al., 2017). Recently, the efficacy of corticosteroids, cyclophosphamide, mofetil/mycophenolate, and calcineurin inhibitors in LN was identified (Fanouriakis et al., 2019), yet low complete response rates, risk of flares, side effects and adverse outcome events of these treatments still remain major concerns to physicians (Hobeika et al., 2019). Hence, finding effective and safe alternative medicines to fight against LN is an urgent global issue to be addressed.

Traditional Chinese medicine is one of the oldest healing systems and has been widely used for thousands of years (Tang et al., 2008; Liu et al., 2021). Due to the multi-targets function (Jiang et al., 2021), traditional Chinese medicine is now recognized as one of the prominent alternative therapies for the treatment of various diseases, including SLE and LN (Yuan et al., 2019a; Zhang and Wei, 2020). Tripterygium wilfordii Hook F. (TWHF) is such a typical representative that has been proved to ameliorate LN through its anti-inflammatory and immunosuppressive effects (Tao and Lipsky, 2000; Ma et al., 2017; Song et al., 2020). However, because of its toxicity, the clinical application of TWHF is severely limited, especially in patients with reproductive needs (Ren et al., 2021). Hedyotis Diffusae Herba (HDH) is another traditional Chinese medicine herb that is widely applied in a variety of prescriptions for the treatment of immune-related diseases (Fan et al., 2010; Jiang et al., 2010). In 2010, Jiang et al. (2010) reported that Bizhongxiao Decoction, which contains HDH, could improve rheumatoid arthritis by regulating the protein expression and function of peripheral blood mononuclear cells. In addition, the protective role and mechanism of HDH in cancer was also demonstrated recently (Wang et al., 2020; Liu et al., 2022; Ma et al., 2023). Besides, Fan et al. (2010) found that the application of HDH could significantly inhibit the expression of regulated on activation, normal T cell expressed and secreted (RANTES, also known as C-C motif chemokine 5), a biomarker of LN (Das and Brunner, 2009), in serum and renal tissue of MRL/lpr mice (lupus-prone mice). However, the specific mechanism of HDH as related to LN remains unclear due to limited researches. Hence, studies investigating key molecular targets and mechanism of HDH against LN needs to be carried out.

Network pharmacology is an emerging interdisciplinary science that integrates virtual computing, high-throughput data analyses, network database retrieval, bioinformatic network construction and network topology analyses (Kibble et al., 2015; Wang et al., 2022a), and was developed for the explanation of the relationship between drug components, targets, and diseases (Luo et al., 2020; Jiao et al., 2022). Nowadays, network pharmacology has become a holistic and efficient tool to unveil the pharmacological mechanism of traditional Chinese medicine and is conducive to provide deep insights into new medicine developing from a network perspective (Hopkins, 2008; Sucher, 2013). HDH, as a traditional Chinese herbal medicine widely applied in prescriptions for LN, has a wide range of pharmacological compounds, however, its mechanism for the treatment of LN remains obscure. In this study, analyses based on integrated network pharmacology were applied to tackle this issue and identify active ingredients and pivotal targets of HDH against LN.

## Materials and methods

### Identifying LN related targets

LN related targets were retrieved from the OMIM database (Hamosh et al., 2000) (https://omim.org/), the DisGeNET database (Piñero et al., 2015; Piñero et al., 2020) (https://www.disgenet.org/) and the DigSee database (Kim et al., 2013; Kim et al., 2017) (http://210.107.182.61/geneSearch/). As a biomedical literature based authoritative database storing information about human genes and genetic phenotypes, OMIM database could provide us with phenotypes related targets. In the OMIM database, the term “Lupus nephritis” was used as a bait to retrieve LN related records and genes, and 75 records containing 35 LN related genes were obtained finally. Distinct with the OMIM database, the DisGeNET database and the DigSee database possess more massive data of human disease-related genes and scoring systems to help screening reliable diseases-related genes. By inputting “Lupus nephritis” into query box and setting “Score_gda” > 0.05 in DisGeNET database or “Evidence Sentence Score” > 0.5 in DigSee database, 51 LN associated targets from the DisGeNET database and 115 targets obtained from the DigSee database were acquired. All genes were then transformed into unified names by UniProt (UniProt Consortium, 2018) (https://www.uniprot.org/). A total of 162 genes were identified as LN targets by removing duplicates.

### Bioactive components of HDH

TCMSP database (Ru et al., 2014) (https://tcmspw.com/tcmsp.php) is a platform that captures relationship between Chinese herbal medicines, targets and diseases. And most importantly, it provides physical and chemical characteristics of herbal ingredients. To obtain bioactive chemical components of HDH, “Hedyotis Diffusae Herba” was used as the search term, and the components meeting following criterions were selected for further analyses: 1) oral bioavailability ≥40%; 2) drug-likeness ≥0.18; 3) number of rotatable bond <10; 4) molecular weight: 180–500; and 5) has corresponding Pubchem Cid. Poriferasterol, stigmasterol and quercetin were identified as bioactive chemical components of HDH.

### Identifying HDH related targets

To acquire HDH related targets, SMILE strings, InChI strings or names of HDH bioactive components that were acquired from the previous step were then inputted into the query boxes of the STITCH database (Szklarczyk et al., 2016) (http://stitch.embl.de/), the BATMAN-TCM database (Liu et al., 2016) (http://bionet.ncpsb.org/batman-tcm/) and the TCMSP database (Ru et al., 2014) (https://tcmspw.com/tcmsp.php), with the results being limited to *homo species*. Targets from the BATMAN-TCM database under the criterion of score >15 were selected for subsequent analyses. And for targets predicted from the TCMSP database, those displayed as originating from the DrugBank database were selected. All the returned targets were converted into standard names by UniProt (UniProt Consortium, 2018). Finally, 77 targets of stigmasterol, 40 targets of poriferasterol and 83 targets of quercetin were predicted as HDH corresponding targets.

### Functional enrichment analyses and network construction

The Gene Ontology (GO) enrichment analysis and Kyoto Encyclopedia of Genes and Genomes (KEGG) pathways enrichment analysis were conducted by online function annotation tool DAVID (Huang et al., 2009a; Huang et al., 2009b) (https://david.ncifcrf.gov/, version 6.8), and further visualized by a data visualization website (http://www.bioinformatics.com.cn/). While the protein-protein interaction (PPI) networks for LN targets, HDH targets and the overlapped targets were constructed by the STRING database (Szklarczyk et al., 2019) (https://string-db.org/, version 11.0), a platform storing known PPIs originating from other databases and predicted PPIs that were obtained through computer algorithms. Further construction of HDH-bioactive components-targets network, LN targets network, and bioactive components-the overlapped targets-enriched KEGG pathways network were conducted by Cytoscape (version 3.7.2).

### Screening of key therapeutic targets

The overlapped targets between LN related targets and HDH related targets were got by using TBtools (Chen et al., 2020a) (version 1.0686). PPI network of these overlapped targets was then retrieved by the STRING database (Szklarczyk et al., 2019), with the species being limited to *homo species* and the confidence level being set to ≥0.900. Based on the PPI network, betweenness centrality plot and degree centrality plot were drawn by using MATLAB software. Finally, pivotal LN related proteins that were targeted by HDH bioactive components were identified by comparing the betweenness centrality score and degree centrality score among the overlapped targets.

### Molecular docking simulation

The crystal structures of these targets were retrieved from PDB database (Berman et al., 2002) (http://www.pdb.org), while 3D-structures of stigmasterol and quercetin were downloaded from Pubchem database (Kim et al., 2023) (https://pubchem.ncbi.nlm.nih.gov/). Molecular pretreatment, molecular docking simulation and the visualization of docking results were performed by PyMOL software (version 2.4.0) and AutoDockTools (Morris et al., 2008; Morris et al., 2009) (version 1.5.6).

## Results

### HDH targets, functions and PPI network

**FIGURE 1:**
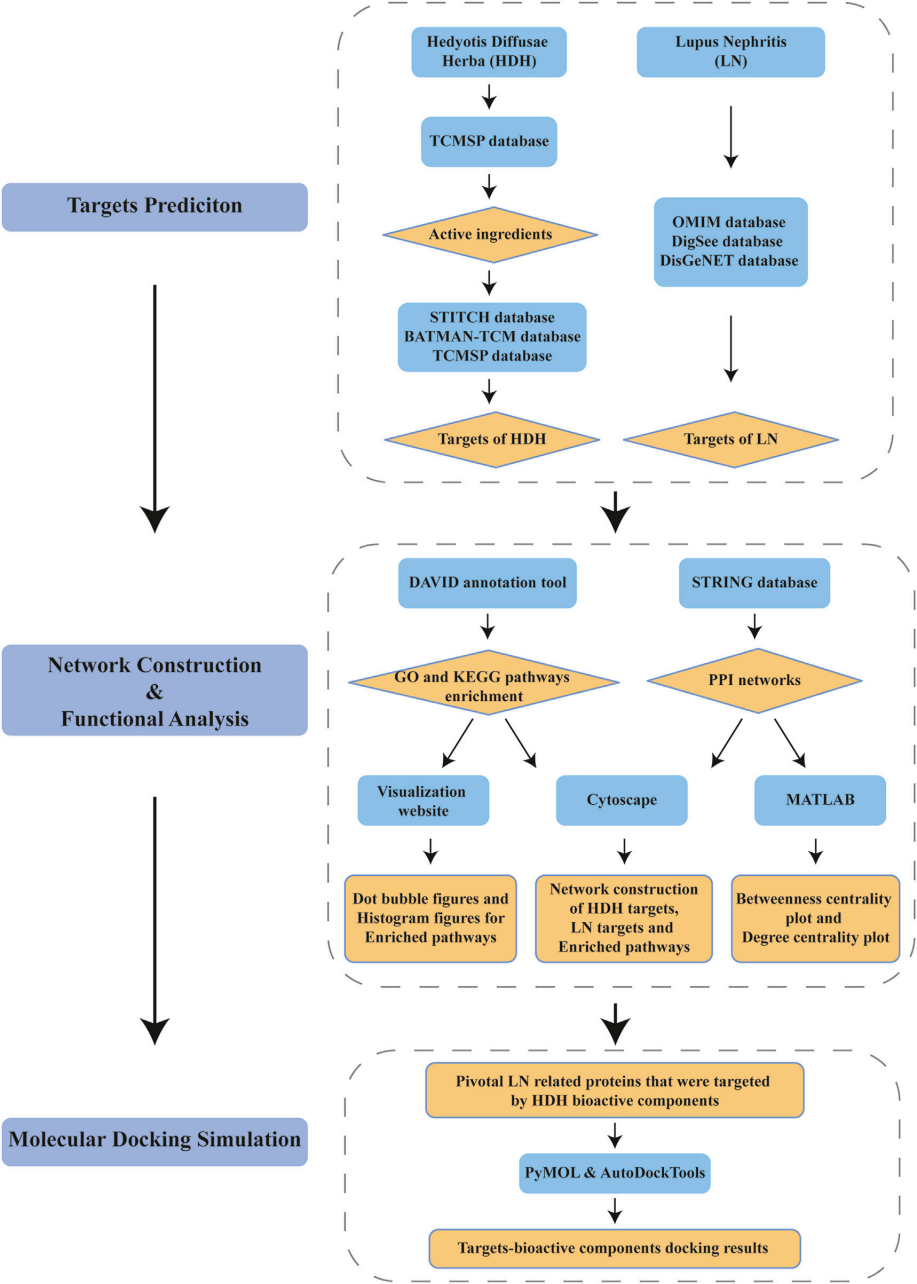
Flow chart of network pharmacology method identifying pivotal targets of Hedyotis Diffusae Herba (HDH) for the treatment of lupus nephritis (LN).

Flow chart of the integrated network pharmacology research is described as in Figure 1. In brief, HDH therapeutic targets and the LN related targets were attained from online databases firstly, with the overlapped part being recognized as latent therapeutic targets of LN targeted by HDH. Subsequently, functional enrichment analyses and PPI network construction were implemented to elucidate the protective mechanism of HDH against LN. Centrality analysis based on the PPI network of shared targets was then performed to help us identify core targets within the network. At last, the binding between core targets and their corresponding bioactive components of HDH was simulated by molecular docking technique.

**FIGURE 2:**
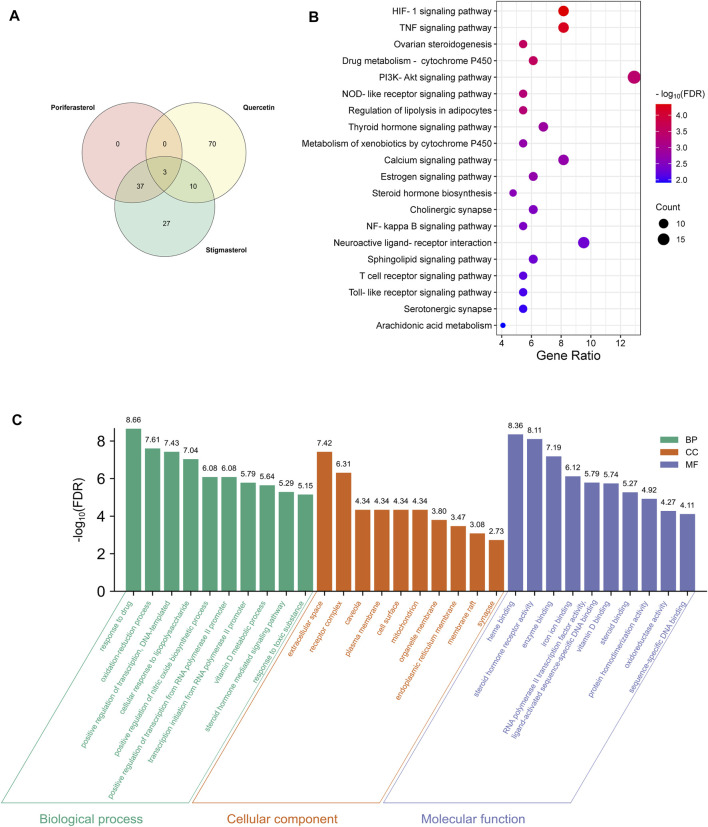
HDH targets and functional enrichment analyses. **(A)** Venn diagram of the predicted HDH targets. **(B)** Dot bubble plot of the top 20 Kyoto Encyclopedia of Genes and Genomes (KEGG) signaling pathways enriched by HDH related targets. The size of dots represents the number of enriched proteins, and the color represents −log_10_ (FDR). **(C)** Histogram plot of the top 10 enriched biological processes, cellular components and molecular functions of HDH targets by Gene Ontology (GO) enrichment analysis. FDR, false discovery rate; BP, biological process; CC, cellular component; MF, molecular function.

**FIGURE 3:**
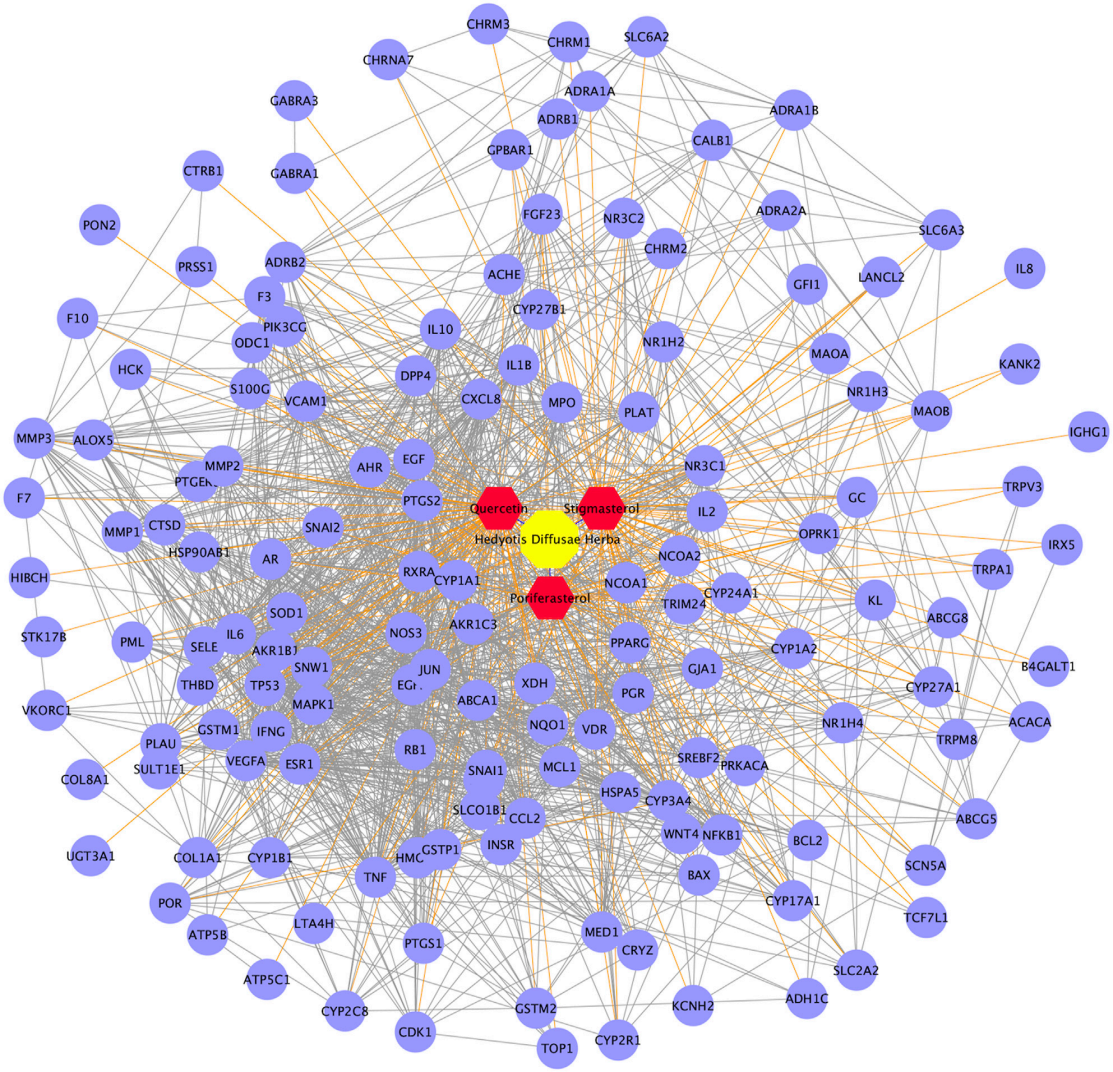
Protein-protein interaction (PPI) network of proteins targeted by three bioactive components of HDH. The yellow octagon represents HDH, the red hexagons represent components of HDH, while the purple dots represent the predicted targets of these three chemical components. The blue lines represent interaction between HDH and its bioactive components, the orange lines represent interaction between HDH bioactive components and the predicted targets, while the PPI networks of targets were represented as prey lines.

Based on the above strategy, three bioactive components of HDH (poriferasterol, stigmasterol, and quercetin) were identified. And an aggregate of 147 targets of these components were predicted by the STITCH database, the BATMAN-TCM database and the TCMSP database (Figure 2A). KEGG enrichment analysis suggested that HDH related targets were mainly involved in HIF-1 signaling pathway, TNF signaling pathway, PI3K-Akt signaling pathway, and NOD-like receptor signaling pathway, etc. (Figure 2B). Besides, as shown in Supplementary Figure S1, HDH corresponding targets were closely related to types of cancers, infectious diseases and autoimmune diseases like rheumatoid arthritis. GO enrichment analysis for cellular component showed that most of these proteins were located in the extracellular space, caveola or plasma membrane, or form receptor complex (Figure 2C). And GO enrichment analysis for biological process indicated that HDH targets might participate in the response to drug, oxidation-reduction process and regulation of transcription (Figure 2C). Besides, the GO enrichment analysis for molecular function implied that targets of HDH active components possessed the ability to bind heme, enzyme and so on; and also had steroid hormone receptor activity, RNA polymerase II transcription factor activity, protein homodimerization activity, oxidoreductase activity, and so on (Figure 2C). Moreover, a PPI network was then constructed for HDH bioactive components related targets (Figure 3). A total of 147 targets nodes and 1,302 protein-protein edges were present in the network of HDH-HDH bioactive components-HDH related targets (Figure 3).

### LN therapeutic targets, functions and PPI network

**FIGURE 4:**
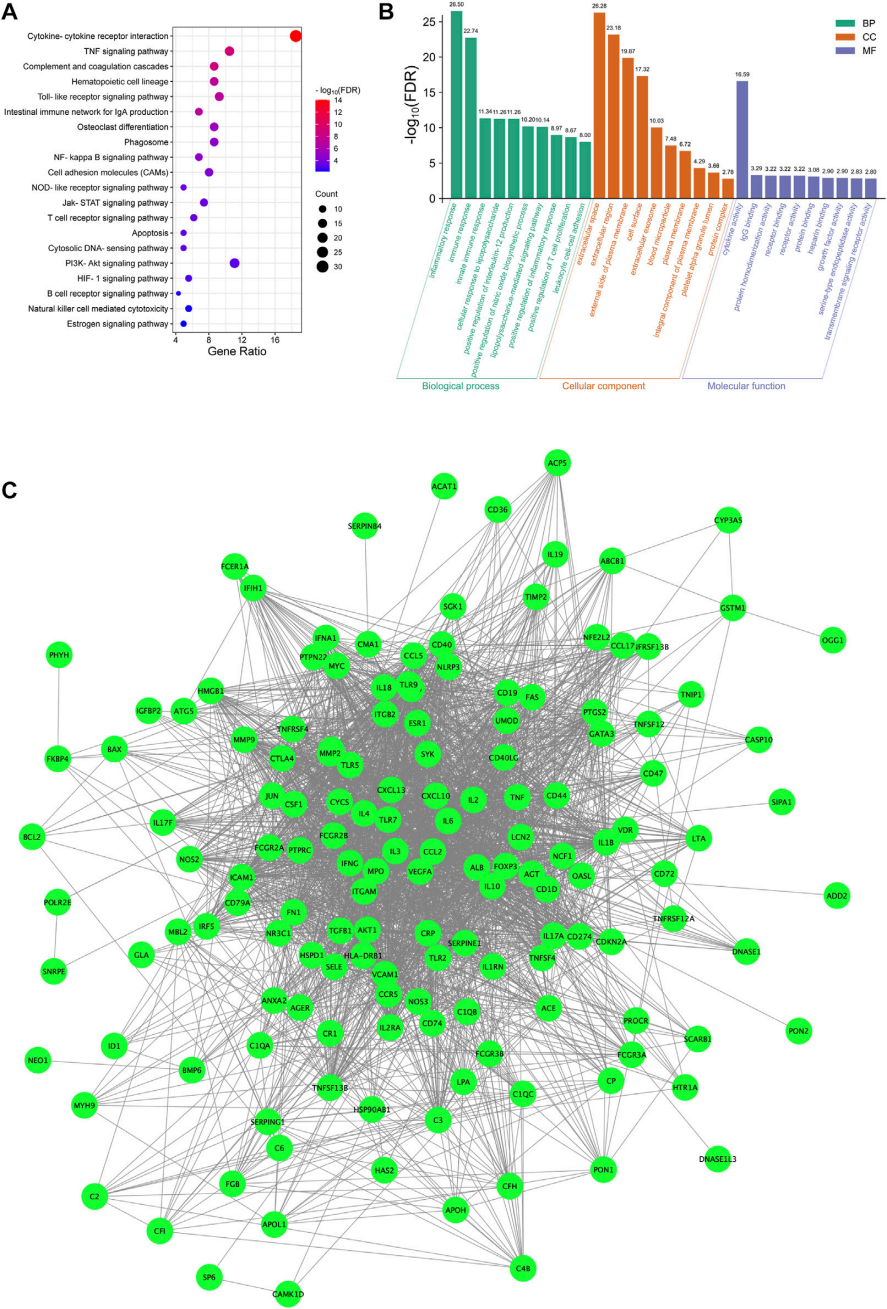
Functional enrichment analyses and PPI network of LN targets. **(A)** Dot bubble plot of the top 20 KEGG signaling pathways enriched by the targets of LN. Size of dots represents the number of enriched proteins, and the color represents −log_10_ (FDR). **(B)** Histogram plot of the top 10 biological processes, cellular components and molecular functions of LN targets by GO enrichment analysis. **(C)** PPI network of LN targets. FDR, false discovery rate; BP, biological process; CC, cellular component; MF, molecular function.

In total, 162 targets were identified as LN therapeutic targets from the OMIM database, the DisGeNET database and the DigSee database. KEGG pathway enrichment analysis illustrated that cytokine-cytokine receptor interaction, TNF signaling pathway, complement and coagulation cascades, Toll-like receptor signaling pathway participated in the course of LN (Figure 4A). Additionally, HDH enriched pathways such as NOD-like receptor signaling pathway, PI3K-Akt signaling pathway, HIF-1 signaling pathway were also enriched in the analysis of LN targets (Figure 4A). On the other hand, besides SLE, these targets were also enriched in other autoimmune diseases like rheumatoid arthritis and inflammatory bowel disease, or infection diseases like tuberculosis, malaria and leishmaniasis (Supplementary Figure S2). GO enrichment analysis for biological process indicated that LN was highly correlated with inflammatory response and immune response (Figure 4B). And as shown in the analysis for cellular component, most of the potential LN therapeutic targets were located in the extracellular space, the external side of plasma membrane and cell surface (Figure 4B). Furthermore, GO enrichment analysis for molecular functions showed that LN targets were enriched in cytokine activity, IgG binding, protein homodimerization activity and so on (Figure 4B). A PPI network was then constructed for LN targets. As described in Figure 4C, these disease targets formed a complex network with 2,701 edges.

### Network and functions of LN-related proteins that were targeted by HDH

**FIGURE 5:**
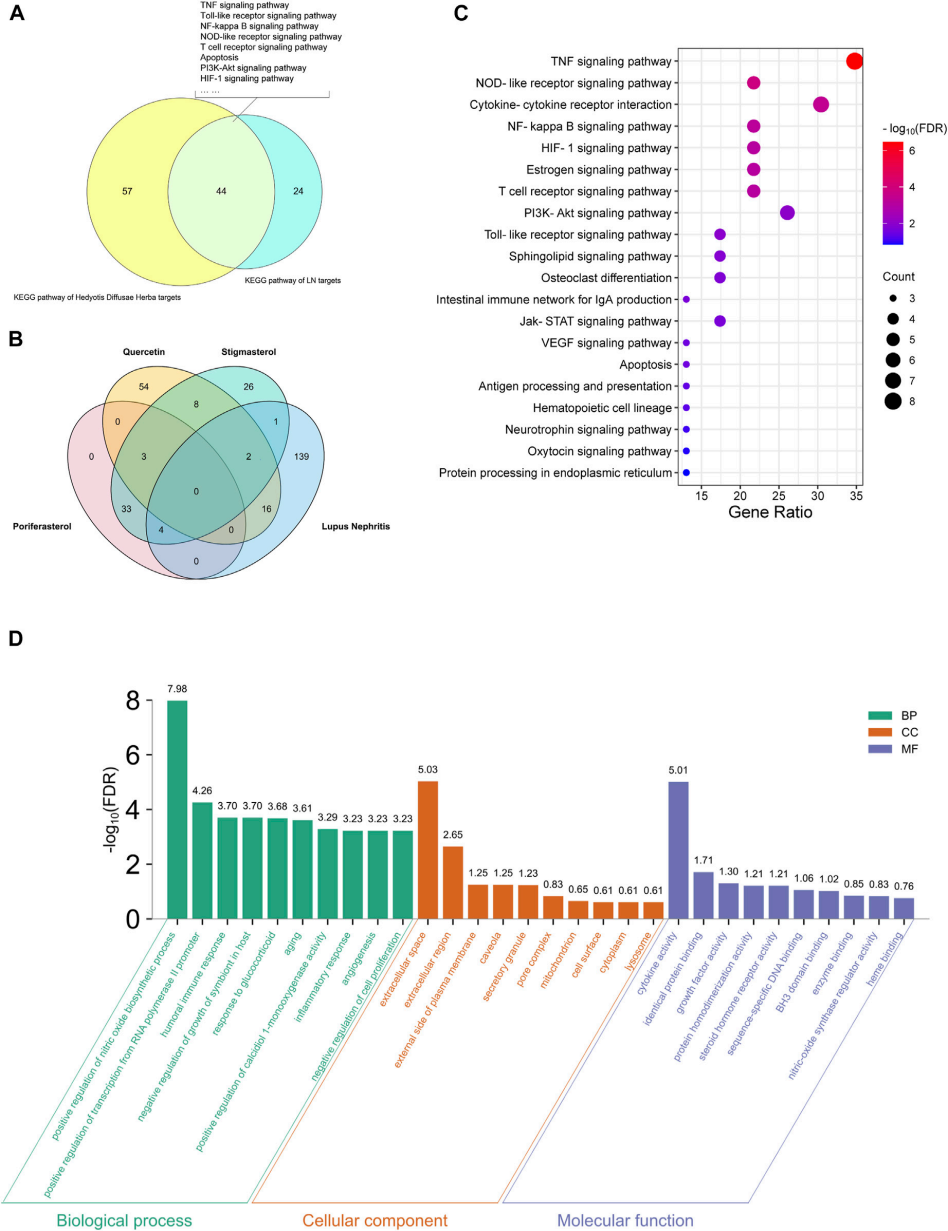
Functional enrichment analyses of LN targets that were targeted by HDH. **(A)** Venn diagram composed of the KEGG pathways of HDH and KEGG pathways of LN targets. **(B)** Venn diagram composed of the predicted poriferasterol targets, the quercetin targets, the stigmasterol targets and the LN targets. **(C)** Dot bubble plot of the top 20 enriched KEGG signaling pathways of the overlapped targets of LN and HDH. The size of dots represents the number of enriched genes, and the color represents −log_10_ (FDR). **(D)** Histogram plot of the top 10 biological processes, cellular components and molecular functions of the shared targets by GO enrichment analysis. FDR, false discovery rate; BP, biological process; CC, cellular component; MF, molecular function.

**FIGURE 6:**
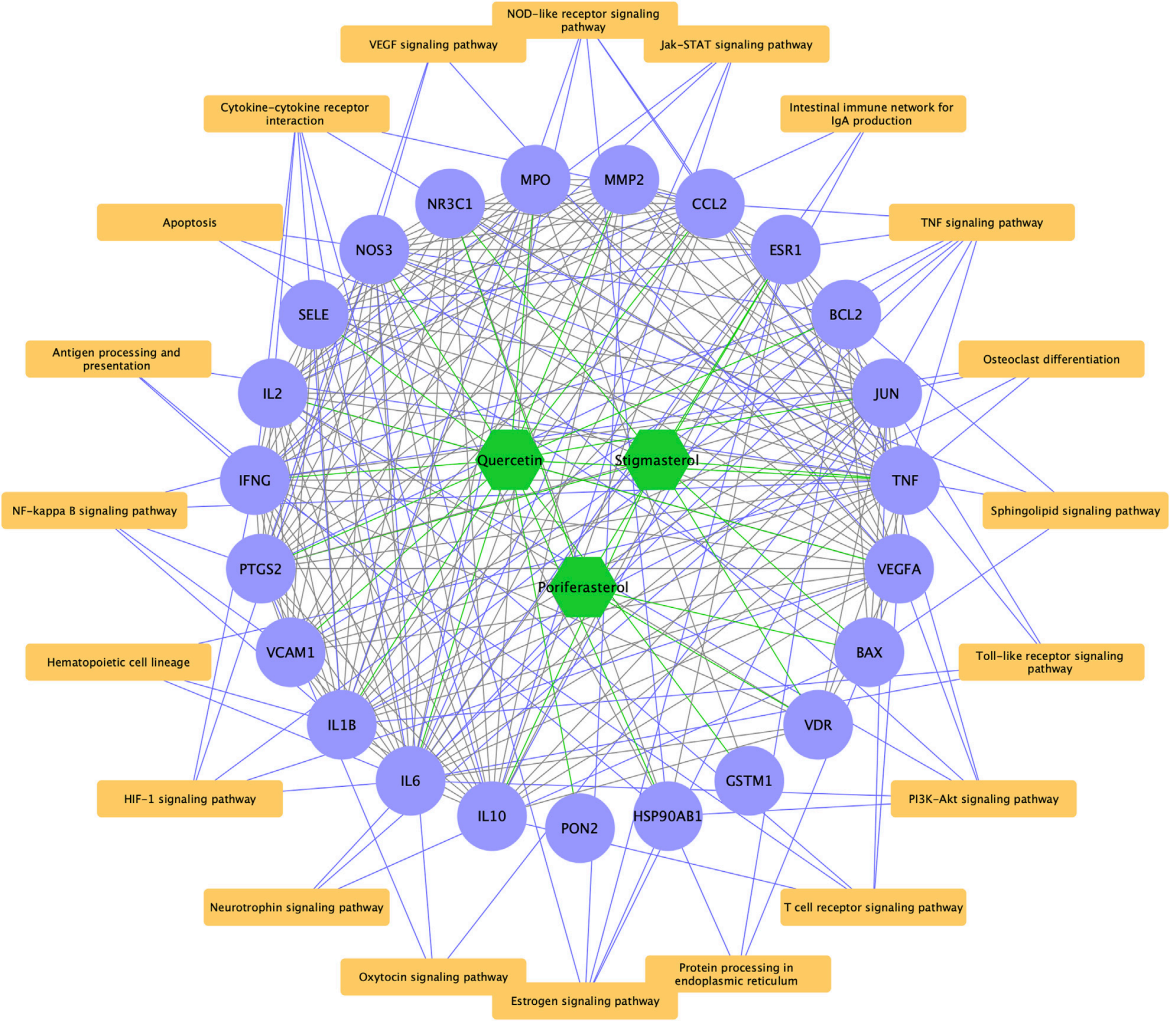
Bioactive components-overlapped targets-KEGG signaling pathways network. The green hexagons represent chemical components of HDH, the purple dots represent the overlapped targets of LN and HDH, while the yellow rectangles represent the top 20 KEGG signaling pathways. The green lines represent interaction between chemical components and the overlapped targets, the gray lines represent the PPI networks, while the purple lines are interaction between the overlapped targets and their enriched KEGG signaling pathways.

As displayed in Figure 5A, 44 pathways were shared between HDH targes and LN targets, including TNF signaling pathway, Toll-like receptor signaling pathway, NF-kappa B signaling pathway and so on. And a total of 23 targets of LN that were also targeted by HDH active components were identified (Figure 5B). Further KEGG enrichment analysis of these shared targets indicated that the functions of these targets were enriched in TNF signaling pathway, NOD-like receptor signaling pathway, cytokine-cytokine receptor interaction, NF-kappa B signaling pathway, and HIF-1 signaling pathway, etc. (Figure 5C). The GO enrichment analysis of these overlapped targets was shown in Figure 5D. The analysis for biological process implied that the overlapped targets were related to the nitric oxide biosynthetic process mostly, and were also associated with humoral immune response (Figure 5D). As for the molecular functions, cytokine activity and identical protein binding were associated with these targets most obviously (Figure 5D). Besides, these targets were located in the extracellular space and external side of plasma membrane mostly (Figure 5D). A network containing the active components of HDH, the overlapped targets and their enriched pathways was displayed in Figure 6.

### Screening of pivotal overlapped targets and molecular docking

**FIGURE 7:**
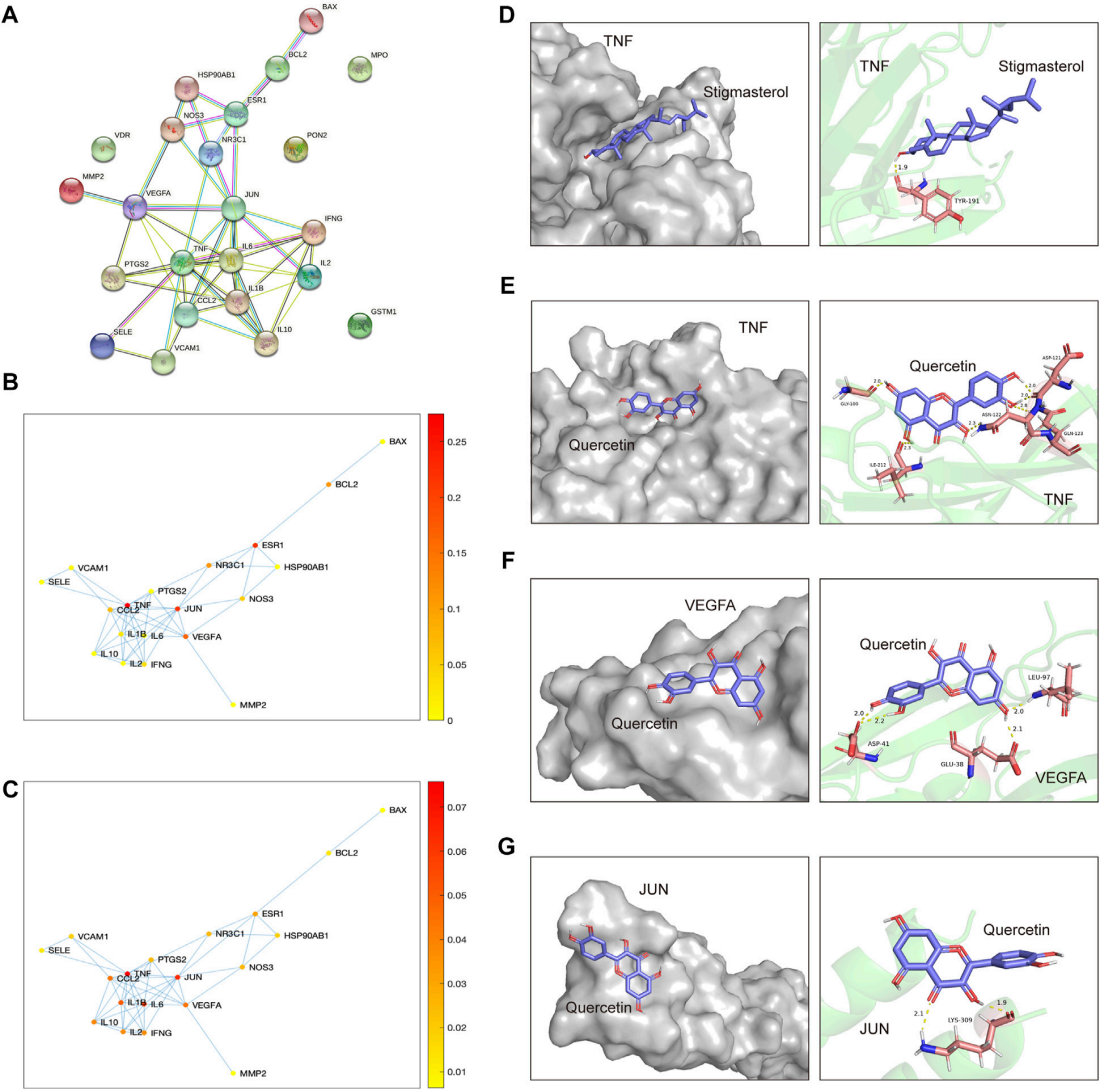
Screening of pivotal overlapped targets and molecular docking of HDH components binding to these pivotal targets. **(A)** PPI network of the overlapped targets with high confidence (interaction score ≥0.900). **(B,C)** Betweenness centrality plot and Degree centrality plot of the interaction network. The color represents the level of the betweenness centrality score or the degree centrality score of every dots. **(D–G)** Molecular models of TNF interacting with stigmasterol **(D)**, TNF interacting with quercetin **(E)**, VEGFA interacting with quercetin **(F)** and JUN interacting with quercetin **(G)**. Small molecule compounds are represented as purple colored, while the gray colored (left panel) and green colored (right panel) macromolecular substances represent the target proteins.

By setting “confidence level” to “≥ 0.900,” a more credible PPI network of the overlapped targets was acquired (Figure 7A). Subsequently, a betweenness centrality map and a degree centrality map based on the predicted PPI network were plotted (Figures 7B, C). According to these maps, TNF, JUN and VEGFA were recognized as the most pivotal targets in the network, as the betweenness centrality score and the degree centrality score of these three targets were highest (Figures 7B, C). Simulation of the binding between these three targets and their corresponding ligands was conducted then, with energy required for binding of each pairs being calculated by AutoDockTools. As summarized in Table 1, binding energy of TNF with stigmasterol was lowest (−6.32 Kcal/Mol), with the binding activity being identified as good. And the free energy required for the binding of TNF with quercetin, and VEGFA with quercetin was −4.56 Kcal/Mol and −4.58 Kcal/Mol, respectively, indicating certain binding activities. While no certain binding activity was found for JUN-quercetin interaction (binding energy =−2.32 Kcal/Mol). Detailed binding modes of macromolecular proteins and their corresponding small ligands were displayed in Figures 7D–G. As shown in Figure 7D, stigmasterol bound with TNF in a groove through 1 hydrogen bond with TYR-191. And Figure 7E implied that quercetin might interact with TNF through six hydrogen bonds with GLY-100, ASP-121, ASN-122, GLN-123 and ILE-212, thus exerting its latent therapeutic effect. Similar results for quercetin with VEGFA and quercetin with JUN could be found in Figures 7F, G.

**TABLE 1:** Energy for binding between pivotal targets and corresponding ligands by AutoDockTools.

Target receptors	Ligands	Binding energy (Kcal/Mol)	Binding activity
TNF	Stigmasterol	−6.32	Good
TNF	Quercetin	−4.56	Certain
VEGFA	Quercetin	−4.58	Certain
JUN	Quercetin	−2.32	—

Note: Binding energy less than −4.25, −5.0, and −7.0 kcal/mol was identified as a certain, good or strong binding activity, respectively.

## Discussion

Traditional Chinese medicine regards individuals as systems with various states, and has accumulated a large number of traditional Chinese medicine prescriptions (Li and Zhang, 2013). Compounds isolated from Chinese herbal medicine, such as antimalarial drug artemisinin, exhibit similar pharmacological activities as pharmaceutical drugs (Sucher, 2013). The exploration of traditional Chinese medicine ingredients will inject new vitality into the development of new drugs (Wang et al., 2018). Recently, the systems pharmacology method has been widely concerned in the field of Chinese medicine (Zhou et al., 2016), facilitating the transformation of traditional Chinese medicine from empirical medicine to evidence-based medicine (Li and Zhang, 2013). Besides, the application of network pharmacology strategy is also attributing to the elucidation of mechanism of Chinese medicine against diseases and the screening of pivotal components of Chinese medicine (Zhou et al., 2016).

In this study, by applying the systematic integrated network pharmacology method to traditional Chinese medicine research, our analyses identified TNF, VEGFA and JUN as the most pivotal therapeutic targets of HDH against LN (Figures 7B, C), with the binding of the former two proteins with their corresponding HDH components being validated by molecular docking, especially the interaction between stigmasterol and TNF (Figures 7D–G, Table 1).

TNF is a cytokine mainly secreted by macrophages, activating the transcription of several proinflammatory genes and is involved in the regulation of a wide spectrum of biological processes such as cell proliferation, differentiation and apoptosis (Wu et al., 2002). Since Jacob et al elucidated the relationship between TNF and LN in 1998 (Jacob and McDevitt, 1988), there is abundant evidence supporting the pathogenicity of TNF in LN (Boswell et al., 1988; Jacob, 1992; Zhao et al., 2013). By enhancing the expression of numerous cytokines, TNF-alpha mediated the recruitment and adherence of pathogenic inflammatory cells in LN, amplifying kidney injury (Wuthrich, 1992; Davidson, 2016). Besides, TNF was also proved to cause direct injury in podocytes (Pedigo et al., 2016). On the other hand, current clinical data shows that the application of TNF blocker induction therapy may lead to long-term remission in patients with LN, although the safety of TNF blockers still needs long-term observation (Aringer and Smolen, 2012). In the current study, we found that the active components of HDH might exert therapeutic effect on LN through TNF signaling pathway. Besides, TNF was identified as a key target in the network constructed for overlapped targets of LN and HDH.

In the current study, we predicted that stigmasterol and quercetin, two bioactive components of HDH, might target TNF and thus influence the function of TNF. Stigmasterol is a plant sterol exhibiting anti-inflammatory activity (Ahmad Khan et al., 2020; Bakrim et al., 2022). Kangsamaksin et al. (2017) reported that stigmasterol could suppress tumor angiogenesis and cholangiocarcinoma growth by inhibiting TNF-α expression. Such effect was also found in collagen induced arthritis (Ahmad Khan et al., 2020). Besides, some common formulas containing stigmasterol possess apparent properties that suppress the expression of TNF-α (Masola et al., 2018; Wang et al., 2022b). However, no research about the protective role of stigmasterol against LN was found until now. We speculate that stigmasterol may possess the ability to bind to TNF in a groove of TNF and then affect the function of TNF, thus ameliorating LN. This hypothesis is worthy of further validation by experiments.

Quercetin is a flavonol widely distributed in plants (Chen et al., 2020b). The effect of quercetin in down-regulating TNF was reported by abundant researches (Yang et al., 2019; Sul and Ra, 2021; Tsai et al., 2021), too. And Liu et al. (2019) further demonstrated that quercetin could block the expression of pentraxin 3 that was induced by TNF-α and inhibit the proliferation of mesangial cells by inhibiting the activation of NF-kappa B signaling pathway, playing a protective role in LN. Nevertheless, the mechanism of reno-protective role of quercetin in LN still needs to be uncovered.

Our analyses also identified VEGFA as a key target in the course of HDH treatment against LN. It was evidenced that the plasma VEGFA level was reduced when SLE patients were treated with Mycophenolate mofetil (Slight-Webb et al., 2019). And Yuan et al. (2019b) concluded that VEGF-endothelin-1 system was involved in the endothelial cell-podocyte crosstalk in LN. However, rare studies have paid attention to the effect of quercetin on VEGFA. Wang and the colleagues discovered that astragalus membranaceus, a Chinese medicine herb containing quercetin, could alleviate acquired hyperlipidemia through regulating lipid metabolism, in which the upregulated VEGFA might be one of the key targets (Wang et al., 2022c). Yu et al. (2022) also found that quercetin could facilitate the upregulation of the expression of VEGFA and improve fat graft survival. But the mechanism of quercetin in the treatment of LN through VEGFA that was predicted in this study still needs to be further studied.

Besides, this study also elucidated that TNF signaling pathway, Toll-like receptor signaling pathway, NOD-like receptor signaling pathway, NF-kappa B signaling pathway, HIF-1 signaling pathway, and PI3K-Akt signaling pathway might be involved in the pharmacological effect of HDH against LN (Figures 4A, 5A, C). TNF signaling pathway (Aten et al., 2000), Toll-like receptor signaling pathway (Pawar et al., 2007), NF-kappa B signaling pathway (Su et al., 2018), HIF-1 signaling pathway (Chen et al., 2020c), and PI3K-Akt signaling pathway (Stylianou et al., 2011) represent classic signaling pathways of LN. However, limited studies interpreted the effect of HDH from these aspects. In the treatment of cervical cancer, researchers found that a traditional Chinese medicine prescription, Yangshe granule that consists of herbs including HDH, exerted anti-cancer effect through regulating the PI3K-AKT signaling pathway and apoptosis (Ma et al., 2023). The above result was consistent with one of the predicted pathways in our study. While all the predicted mechanism needs to be further validated by experiments.

In general, our study mainly found that the active components of HDH, stigmasterol and quercetin, may play a protective role in LN patients by acting on TNF and VEGFA. Yet the interaction predicted in this study still needs to be tested *in vitro* and *in vivo*. However, it should be emphasized that even though the study identified several key targets, the multi-targets effect and multi-pathways effect of traditional Chinese medicine cannot be ignored, and the therapeutic effects of HDH in LN cannot be simply attributed to these binding. Anyway, the research provides us new insights into the therapeutic mechanism of HDH in LN. And the network pharmacology methods used in this study also offered guidance and inspiration for further researches and the development of new drugs.

## Data Availability

The original contributions presented in the study are included in the article/Supplementary Material, further inquiries can be directed to the corresponding author.
