# Supplementary material for: Molecular mechanism of Hedyotis Diffusae Herba in the treatment of lupus nephritis based on network pharmacology
Source: Front Pharmacol. 2023 Jun 8;14:1118804. doi: 10.3389/fphar.2023.1118804 (PMC10285311; doi:10.3389/fphar.2023.1118804)
Supplement: Supplementary file 1 [file DataSheet1.PDF]

Supplementary Figures

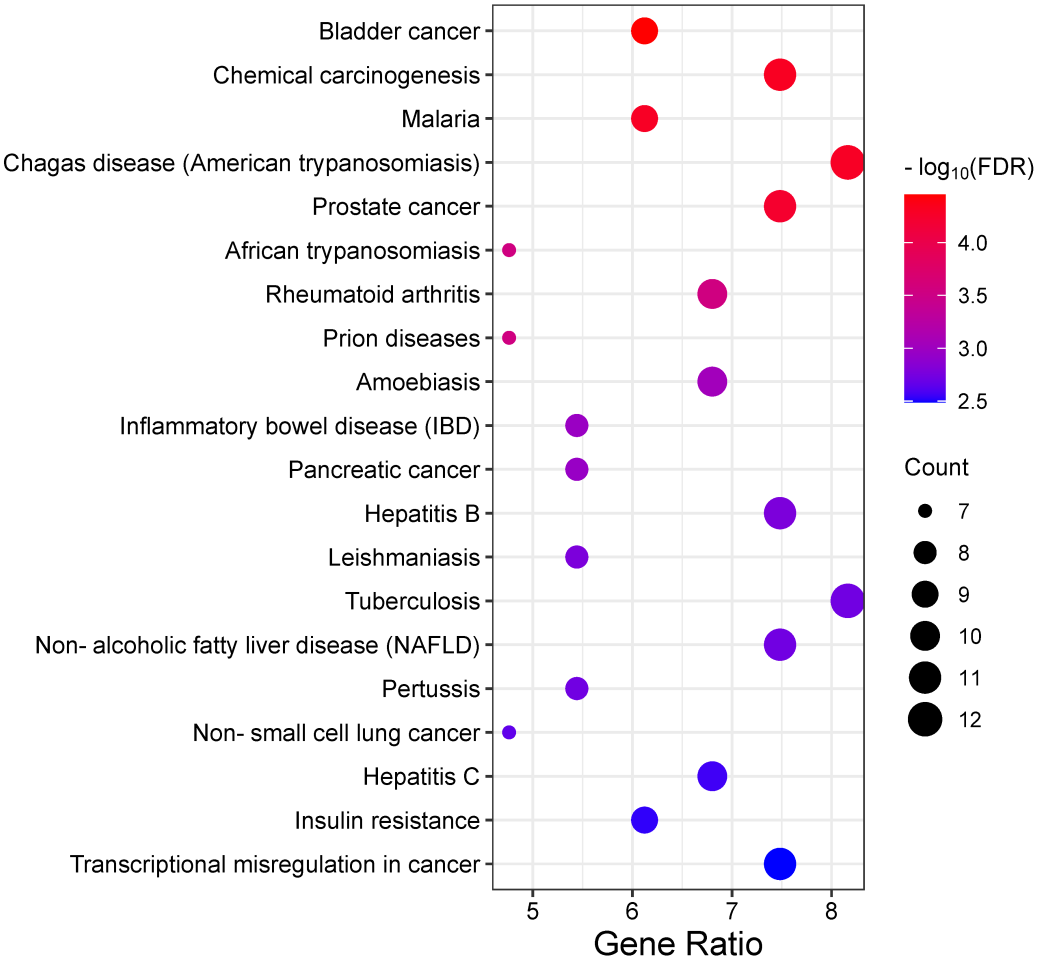

Supplementary Figure 1. Dot bubble plot of the top 20 diseases enriched by Kyoto Encyclopedia of Genes and Genomes (KEGG) enrichment analysis of HDH related targets. The size of dots represents the number of enriched proteins, and the color represents $-\log_{10}$ (FDR). FDR: false discovery rate.

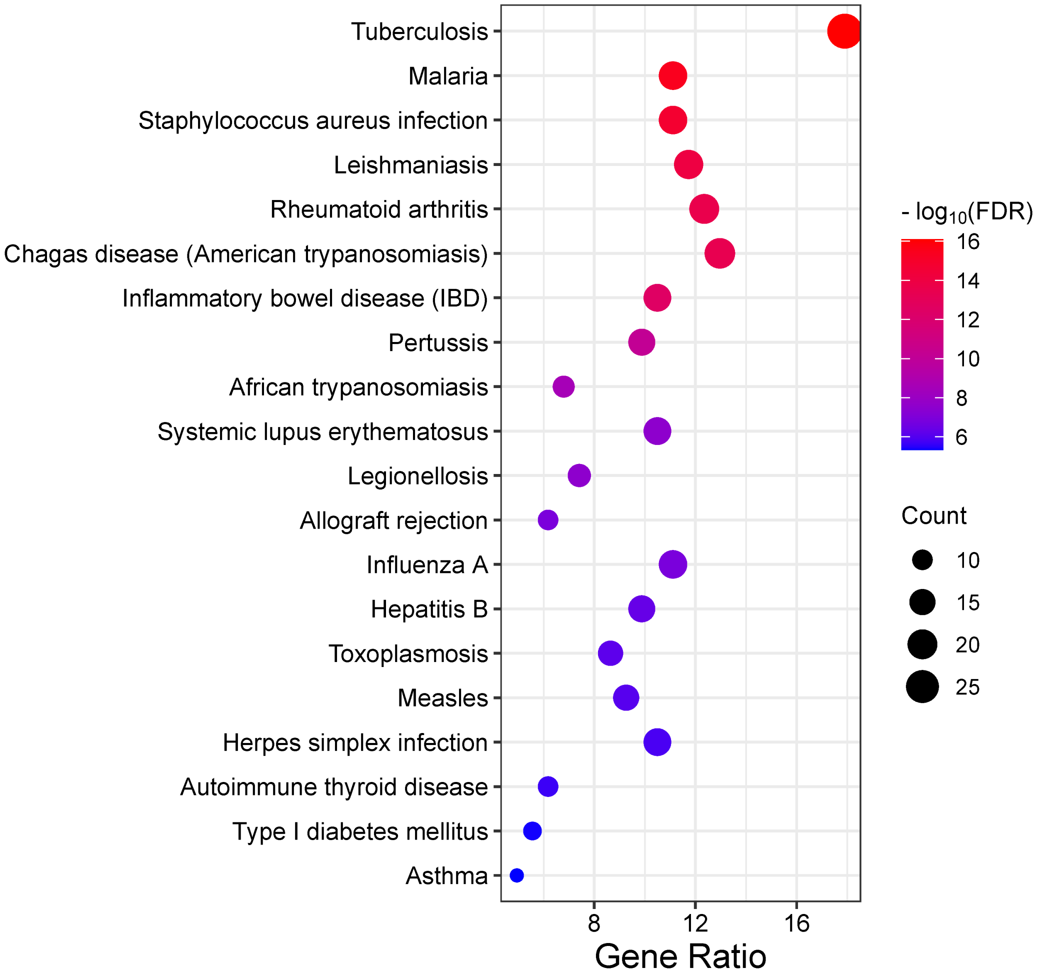

Supplementary Figure 2. Dot bubble plot of the top 20 diseases enriched by KEGG enrichment analysis of the targets of LN. The size of dots represents the number of enriched proteins, and the color represents $-\log_{10}$ (FDR). FDR: false discovery rate.
